# Supplementary material for: Universal and quantitative detection of double-stranded RNAs as a signature of pan-virus infections using a luciferase-based biosensor
Source: J Biol Eng. 2025 Dec 16;20:11. doi: 10.1186/s13036-025-00601-0 (PMC12822233; doi:10.1186/s13036-025-00601-0)
Supplement: Supplementary file 1 — Supplementary Material 1 [file 13036_2025_601_MOESM1_ESM.pdf]

Supplementary Material

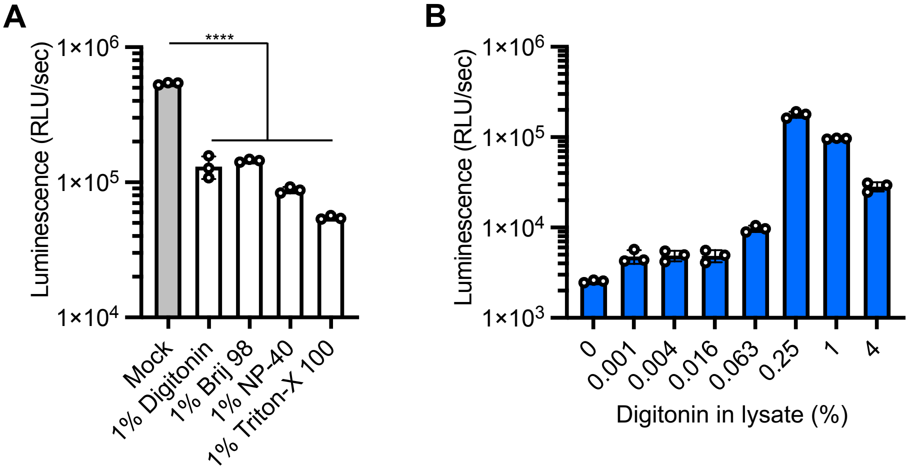

Figure S1. Negative impact of detergent contamination on the luciferase assay with the dsRNA biosensor **(A)** RNA samples extracted from JEV BHK-21 cells at 48 hpi with JEV were mixed with the indicated detergents at a final concentration of 1% and then analyzed by the luciferase assay for dsRNA detection with the dsRNA biosensor. **(B)** Cells at 48 hpi following JEV infection were suspended in lysis buffer containing different concentrations of digitonin and analyzed by the luciferase assay with the dsRNA biosensor. The values shown are mean ± SD of triplicate samples. ****$p < 0.0001$ by one-way ANOVA with Dunnett's test (A).

Supplementary materials

Table S1. Primers used for virus RNA quantification in this study

Primer Name	Sequence (5' to 3')	Reference
JEV NS2A-F	AGCTGGGCCTTCTGGT	[49]
JEV NS2A-R	CCCAAGCATCAGCACAAG	
JEV NS2A-Probe	FAM- CTTCGCAAGAGGTGGACGGCCA-BHQ1	
Mie41(10123)-F	CAAGTCTGGAACAGGGTATGG	This study
Mie41(10356)-R	TCTTCTGAGGGAAGTCATGTAATC	
Mie41(10582)-F	CGCACCGGAAGTTGAAAGAC	
Mie41(10770)-R	TGTTGTTTCCACGGGGTCTC	
Scrambled sfRNA-F	AAAGCCACCTGTATCTAGGGTA	
Scrambled sfRNA-R	AGCACCGTCTCATGTTTATGG	
Ham_Actb-F	ACTGCCGCATCCTCTTCCT	[50]
Ham_Actb-R	TCGTTGCCAATGGTGATGAC	
Ham_Actb-Probe	FAM-CCTGGAGAAGAGCTATGAGCTGCCTGATG-BHQ1	

Table S2. Primers used for ITV template DNA preparation

Primer Name	Sequence (5' to 3')	Product
T7-sfRNA-F	ATTTAATACGACTCACTATAGGGTGGAGTCAGGCCAG CAAAAGCTG	JEV sfRNA
T7-sfRNA-R	AGATCCTGTGTTCTTCCTCACCACCAGCTA	
T7-Scr-F	ATTTAATACGACTCACTATAGGGACCGACTCG	Scrambled sfRNA
T7-Scr-R	ATCTTAGTGCGGGCTTACGCTCCTCTGA	
T7-CDS(525)-F	ATTTAATACGACTCACTATAGGGCCGTGCAGAGGGCA GGATGAGCTGA	CDS(525)
T7-CDS-R	CTAAATAACCCTGTCCTCCTGAATTAATACATC	
T7-CDS(3000)-F	ATTTAATACGACTCACTATAGGGCTGGAAAGAACTAC TCCTTTGATGCAGA	CDS(3000)
T7-CDS-R	CTAAATAACCCTGTCCTCCTGAATTAATACATC	
T7-AcGFP-F	ATTTAATACGACTCACTATAGGGATGGTGAGCAAGGG CGCCGAGCTGT	AcGFP
T7-AcGFP-R	CTTGTACAGCTCATCCATGCCGTG	

Table. S3. Nucleotide sequence of IVT template of scrambled sfRNA

Name	Sequence (5' to 3')
T7-Scrambled sfRNA	ATTTAATACGACTCACTATAGGGACCGACTCGCGATCCACGTAGAAGGGCATTA<br>AAGGCCGCGGCACCTGTATGTAGATCGTACAGACAGAAGAATTGACATAGCCA<br>TGGTATTAAGTTGGATGGGTACGCACTCCCGGGCCCGCGGGAGGGCACCAAGA<br>GCTACAATAGACGCAACCGGCTCTGTAAAAGCCACCTGTATCTAGGGTACCTAG<br>GGAAGACGCACGGGCCCCACTTCGAGTACCTCTCACGCCGGCAAGGGTTTTGA<br>TCGATGGGAAACCCGGAGTAGACGCATAGGAGGCCAGCGCGAGCACGAAGGC<br>AAAGGCTGAGTCCCCAGCTAACCAAGGCAGCCATGTAACTTCGGCTGCCATAA<br>ACATGAGACGGTGCTTCGCAGTCGCTTACGGGGCCCAGAACTTTCAGGCTGAA<br>AAAACTATGTGAAGGGAGAGCAGCGTGTTTTCGGTGGATGAGGACGAGCAAG<br>CTTGATCGGGTCCGGATCCTCGTGCCATTAGAGGAGGCCTAATCAGAGGAGCGT<br>AAGCCCGCACTAAGAT
